# Fiberoptic‐guided tracheal intubation under precise anesthesia and topicalization with spontaneous respiration preservation for an uncooperative patient with severe postburn mentosternal contracture

**DOI:** 10.1002/ccr3.5208

**Published:** 2021-12-07

**Authors:** Zhi Wang, Yong Yang, Yang Chen, Bin Yi, Kaizhi Lu, Bing Chen

**Affiliations:** ^1^ Department of Anesthesia Southwest Hospital Army Military Medical University Chongqing China; ^2^ Department of Anesthesia The Second Affiliated Hospital of Chongqing Medical University Chongqing China

**Keywords:** awake intubation, difficult airway, sevoflurane, spray‐as‐you‐go

## Abstract

Airway management of patients with difficult airways is a challenge to the anesthesiologists and awake tracheal intubation is the recommended strategy. Successful fiberoptic‐guided tracheal intubation under precise anesthesia and topicalization with spontaneous respiration preservation was achieved in an uncooperative patient with severe postburn mentosternal contracture scheduled for release of contracture.

## Graphical abstract

Fiberoptic‐guided tracheal intubation under precise anesthesia and topicalization with spontaneous respiration preservation can be considered in uncooperative patients with severe postburn mentosternal contracture scheduled for release of contracture.

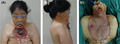

## 1 INTRODUCTION

Airway management of patients with postburn mentosternal contracture is a challenge to the anesthesiologists.^1^, ^2^ Awake tracheal intubation (ATI) has been recommended as the gold standard in airway management for anticipated difficult airway.^3^ However, ATI needs patients' cooperation and may leave a discomfort and nociceptive recall to patients, which leads to the rejection of ATI in the next surgery. We described a patient with severe postburn contracture who refused ATI due to prior unpleasant and unsuccessful multiple tracheal intubation attempts. Successful fiberoptic‐guided tracheal intubation under precise anesthesia with preservation of spontaneous respiration and precise topicalization of airway using a modified spray‐as‐you‐go technique was achieved.

## 2 CASE PRESENTATION

**FIGURE 1:**
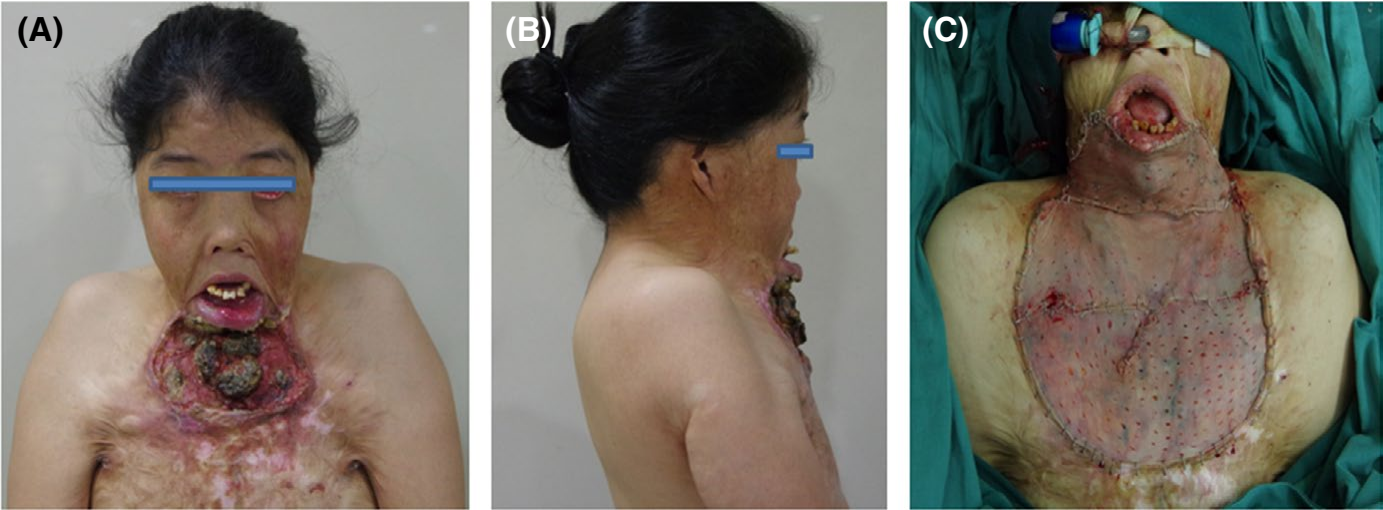
Face, neck, and chest appearance of a 33‐year‐old woman with severe postburn mentosternal contracture. Views on the front (A) and lateral (B) showed obvious contractures and a large carcinoma on the face, neck, and chest, and a small oral opening. (C) Intubated state after skin grafting surgery

A 33‐year‐old woman, with severe postburn mentosternal contracture and cicatricial carcinoma, presented for skin grafting surgery in our hospital. The burn occurred when she was 4‐year‐old. She underwent two reconstructive procedures at 7 and 14 years in local medical centers. Due to the pain caused by the occurrence of cicatricial carcinoma, affecting eating and speaking, she had tried several medical centers for treatment in the past year, but failed for unsuccessful ATI. In preoperative physical examination, severe scar contractures and large tumor of approximately 15 × 12 cm were observed on the lower lip, neck, and anterior chest (Figure 1A and B); the chin, chest, and bilateral armpits fused together; the cervicomental and mentosternal angles completely obliterated; the anterior neck structures, including the larynx, the trachea, and the carotid arteries, were unidentifiable or impalpable. Mouth opening was limited (15 mm) and Mallampati test could not be performed. The left nostril was obstructive for stenosis, but the right nostril breathing was smooth. Preoperative X‐rays and a computed tomography scan (data not shown) revealed distortion of the upper airway and no stenosis of the trachea. It was difficult to perform face mask ventilation because of the nearly fixed neck and regressed mandible.

According to the guidelines on the management of difficult airway,^3^ awake flexible bronchoscopic intubation with topicalization is preferred in such patients, but the patient rejected ATI for discomfort and nociceptive recall before. Meanwhile, other awake strategies, including lightwand, GlideScope^Ⓡ^ Video laryngoscope, laryngeal mask airway, oral or nasal blind intubation, retrograde intubation, surgical tracheostomy, seem impossible. Therefore, a flexible bronchoscopic intubation protocol under precise sedation, topicalization, and spontaneous respiration preservation seems a promising strategy, but the airway should be secured for there was no definite backup plan.

**FIGURE 2:**
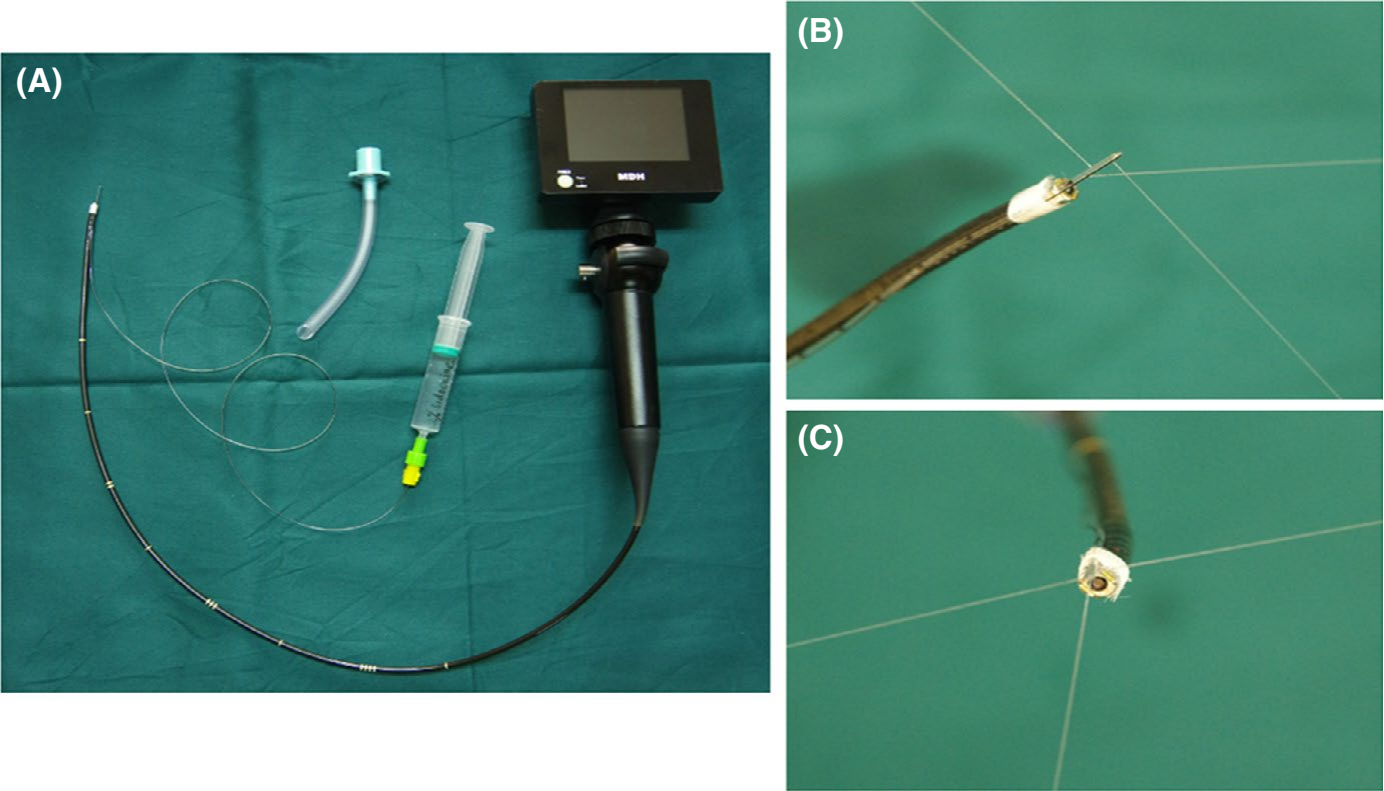
Modified spray‐as‐you‐go technique. (A) An epidural catheter was fixed at the end of the fiberscope. Supplemental oxygen was delivered via a tube through the mouth to avoid hypoxemia. Views on the lateral (B) and top (C) showed the local anesthetic was sprayed out from the epidural catheter

**FIGURE 3:**
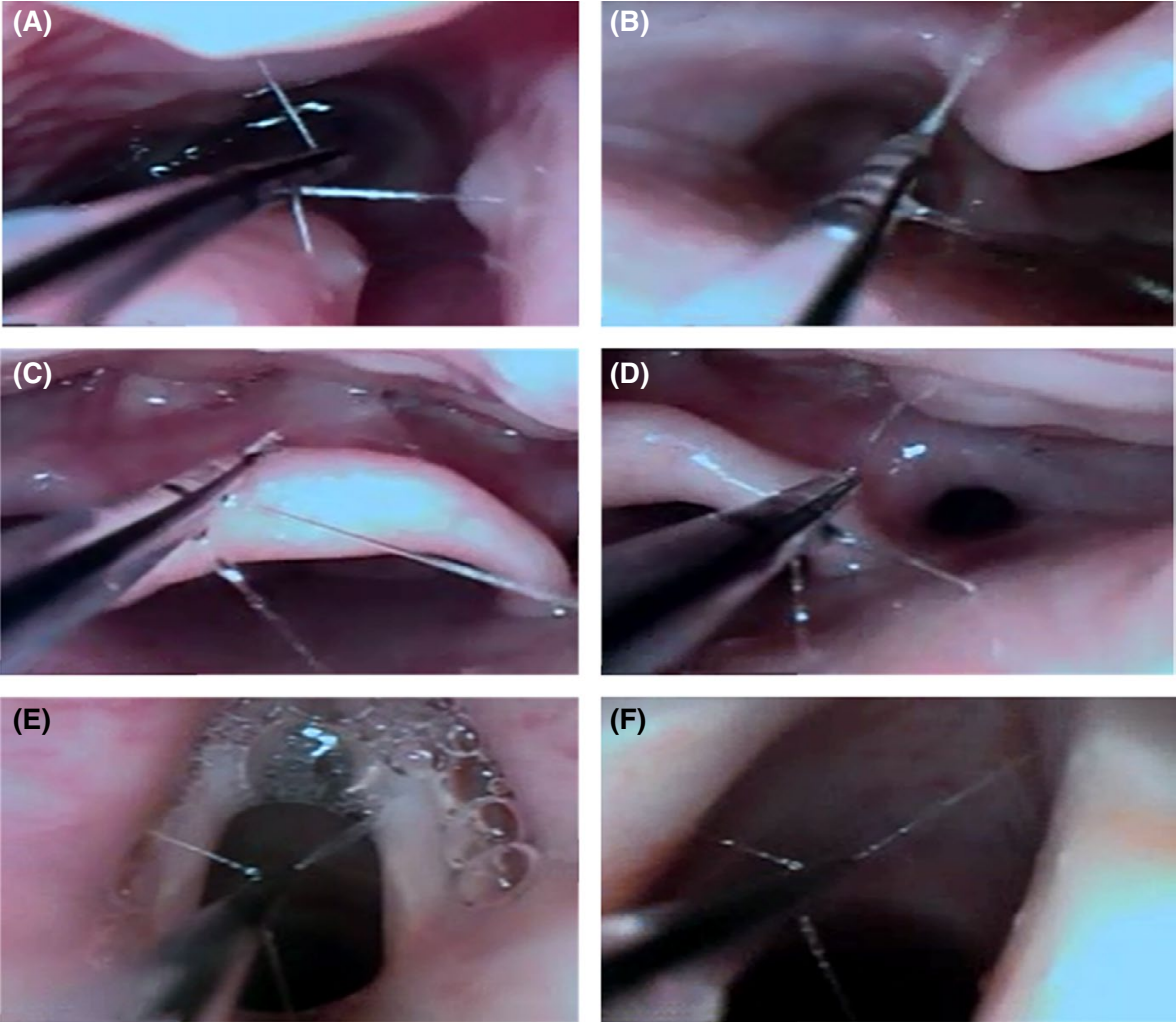
Images showed the anesthesiologist sprayed 2% lidocaine via an epidural catheter onto the patient's airway mucosa. (A) Posterior nasal canal, (B) left pharyngeal recess, (C) epiglottis, (D) right pharyngeal recess, (E) supraglottis, (F) subglottis, and trachea

A written informed consent was taken with explanation focused on the risks of difficult airway. Atropine 0.5 mg was intramuscular injected to reduce secretion 30 min before transferring to the operating room. The patient was monitored by respiration rate, ventilation volume, pulse oximetry, electrocardiogram, blood pressure and bispectral index (BIS), and preoxygenated once she arrived in the operation room. Vital signs were stable. A pillow and some folded sheets were stuffed between the patient back and the operating bed to make her feel comfortable. Intravenous midazolam 2 mg and sufentanil 5 μg were given. Nasal passage was anesthetized by pledgets with 2% lidocaine and 0.25% phenylephrine. Sevoflurane 2% with a flow rate of 4 L/min with 100% O_2_ was administered via an endoscopic mask connected to the ventilator circuit. The concentration of sevoflurane was gradually increased to 4%^4^ and stopped when the BIS reached 55–60 within 3 min, then airway reassessment and precise topicalization were achieved by a modified SAYGO technique.^5^, ^6^, ^7^ An epidural catheter (Figure 2), with an outer diameter of 3.8 mm, was fixed and 1.5 cm longer was applied at the end of the fiberscope (MDH A10; Zhuhai Mindhao Medical Technology Co., Ltd., ShenZhen, China). Topicalization was achieved by spraying 2% lidocaine (7 ml) via the catheter onto the posterior nasal canal, pharyngeal cavity, epiglottis, glottis, and tracheal (Figure 3). Meanwhile, supplemental oxygen 10 L/min was delivered by a hose (Figure 2A) through the mouth to avoid hypoxemia. When lidocaine worked, about 5 minutes later, sevoflurane was inhaled again until the BIS reached 55–60. Then, a Parker Flex‐Tip^®^ tube with an inner diameter of 6.0 mm (Lead Medical Instrument Co., Ltd., Guangzhou, China), which was heated by 40 ℃ and lubricated in advance, was successfully and smoothly inserted into the trachea from the direction of flexible bronchoscopy (Figure 1C). No stress reactions, including cough, hypertension, tachycardia, arrhythmia, hypoxia, and bronchospasm, and no intubation related complications, such as airway trauma, airway obstruction, and bleeding, were occurred during intubation. Finally, the patient was very satisfied with this experience after emergence.

## 3 DISCUSSION

General recommendations for difficult airway management are awake fiberoptic intubation, awake video laryngoscope, laryngeal mask airway as an intubating conduit, lightwand, oral or nasal blind intubation, retrograde intubation, invasive airway access, and ECMO which is the last resort.^8^ In this case, due to the severe contractures and microstomia, direct visualization of the pharynx and larynx by video laryngoscope seemed impossible. Laryngeal mask airway was not considered because size # 3 or 4 for a female adult is too large to pass through her mouth. Orotracheal intubation with lightwand was excluded on the reason that neck scars cannot be illuminated. Retrograde intubation and tracheostomy were also excluded for the reason that anterior neck structures, including the larynx, trachea, and carotid arteries, are unidentifiable and impalpable. For the distortion of the upper airway and uncertainty of success, oral or nasal blind intubation is not an option, except as a "desperate" measure. Due to a good safety and success profile, awake fiberoptic intubation is a preferred choice of anticipated difficult tracheal intubation.^3^ However, ATI may give patients significant discomfort and nociceptive recall^9^, ^10^, ^11^ or be perceived as potentially dangerous when causing a pronounced sympathetic response.^12^ Furthermore, due to the several failures of awake intubation in other medical centers, the patient refused to accept it. Therefore, fiberoptic intubation with precise sedation, topicalization, and spontaneous respiration preservation seems an ideal choice.

At present, many sedative drugs, such as midazolam, dexmedetomidine, propofol, sevoflurane, can be selected. A low dose of midazolam produces a direct amnesia effect. Dexmedetomidine provides a good sedative effect in awake conditions, but the sedation needs to be carefully titrated as excessive sedation can lead to hypoventilation and bradycardia, or inadequate sedation leads to discomfort, anxiety and excessive sympathetic discharge. Moreover, dexmedetomidine may cause nociceptive recall.^7^, ^13^ Compared with propofol intravenous anesthesia, sevoflurane inhalational has less effect on respiratory depression.^12^ Moreover, sevoflurane can be quickly washed out compared with intravenous drugs. Thus, it is more controllable than other intravenous drugs. In this case, midazolam 2 mg, sufentanil 5 μg, and intermittent sevoflurane inhalation were administrated and BIS was monitored to provide precise sedation. Meanwhile, an oral hose was connected to a high flow of oxygen to prevent hypoxia. However, sedation should not be used as a substitute for inadequate airway topicalization.^3^

To sufficiently anesthetize the upper airway and suppress the gag, swallow and cough reflexes, a precise topicalization is essential. There are many ways of airway topicalization, such as nebulization with lidocaine, nerve block, thyrocricocentesis spraying, and the SAYGO technique.^2^ However, research indicated that atomization of local anesthetics had a potential possibility of higher stress responses and poisoning.^14^ Due to anatomical structure changes, nerve block and thyrocricocentesis were impossible for such kind of patients. Compared with the classical SAYGO technique that lidocaine sprayed directly via the working channel of the fiberscope, the modified SAYGO technique, which lidocaine sprayed via an epidural catheter inserted through the working channel of the fiberscope, controls the dosage of local anesthetics more accurately and does a better anesthesia effect, therefore usually used in patients with difficult airway.^5^, ^6^, ^7^ However, the epidural catheter does not match with all fiberscopes. Here, we directly fastened the epidural catheter at the end of the fiberscope. Moreover, this SAYGO technique can evaluate the airway passage directly, which increases the safety of intubation. Thus, it seemed a better choice in this case.

Furthermore, once the local anesthetics worked, the patient was sedated by sevoflurane inhalation again, then intubated by the guidance of flexible bronchoscopy. Taken together, a safe fiberoptic‐guided nasotracheal intubation under precise anesthesia with spontaneous respiration and precise topicalization of the airway by a modified SAYGO technique was achieved in a patient of severe difficult airway who was unwilling for ATI. Moreover, this strategy is not limit to postburn patients with difficult airway.

## 4 CONCLUSION

This case demonstrates that precise anesthesia with preservation of spontaneous respiration and precise topicalization of the airway by a modified SAYGO technique provide a safe and successful fiberoptic‐guided tracheal intubation in uncooperative patients with an anticipated difficult airway, such as severe postburn mentosternal scar contracture and/or unwillingness to accept ATI.

## CONFLICT OF INTEREST

None.

## AUTHOR CONTRIBUTIONS

ZW, YY, and BC: study conception and design, acquisition, analysis, and interpretation of data, drafting and revising the article; YC, BY, KL: revising the article. All authors read and approved the final manuscript and agreed to be accountable for all aspects of the work.

## CONSENT

A written consent was obtained from the patient for the publication of the case and images.

## Data Availability

No data were used during the study.
